# Particulate matter components and subclinical atherosclerosis: common approaches to estimating exposure in a Multi-Ethnic Study of Atherosclerosis cross-sectional study

**DOI:** 10.1186/1476-069X-12-39

**Published:** 2013-05-03

**Authors:** Min Sun, Joel D Kaufman, Sun-Young Kim, Timothy V Larson, Timothy R Gould, Joseph F Polak, Matthew J Budoff, Ana V Diez Roux, Sverre Vedal

**Affiliations:** 1 Department of Environmental and Occupational Health Sciences, University of Washington School of Public Health, 4225 Roosevelt Way NE, #100, Seattle, WA, 98105, USA; 2 Department of Occupational Health, Tianjin Medical University School of Public Health, Tianjin, China; 3 Department of Medicine, University of Washington School of Medicine, Seattle, WA, USA; 4 Department of Epidemiology, University of Washington School of Public Health, Seattle, WA, USA; 5 Department of Civil and Environmental Engineering, University of Washington College of Engineering, Seattle, WA, USA; 6 Department of Radiology, Tufts University School of Medicine, Boston, MA, USA; 7 Department of Medicine, University of California David Gelfen School of Medicine at Los Angeles, Los Angeles, CA, USA; 8 Department of Epidemiology, University of Michigan School of Public Health, Ann Arbor, MI, USA

**Keywords:** Atherosclerosis, Cardiovascular diseases, Coronary artery disease, Air pollution, Particulate matter

## Abstract

**Background:**

Concentrations of outdoor fine particulate matter (PM_2.5_) have been associated with cardiovascular disease. PM_2.5_ chemical composition may be responsible for effects of exposure to PM_2.5_.

**Methods:**

Using data from the Multi-Ethnic Study of Atherosclerosis (MESA) collected in 2000–2002 on 6,256 US adults without clinical cardiovascular disease in six U.S. metropolitan areas, we investigated cross-sectional associations of estimated long-term exposure to total PM_2.5_ mass and PM_2.5_ components (elemental carbon [EC], organic carbon [OC], silicon and sulfur) with measures of subclinical atherosclerosis (coronary artery calcium [CAC] and right common carotid intima-media thickness [CIMT]). Community monitors deployed for this study from 2007 to 2008 were used to estimate exposures at baseline addresses using three commonly-used approaches: (1) nearest monitor (the primary approach), (2) inverse-distance monitor weighting and (3) city-wide average.

**Results:**

Using the exposure estimate based on nearest monitor, in single-pollutant models, increased OC (effect estimate [95% CI] per IQR: 35.1 μm [26.8, 43.3]), EC (9.6 μm [3.6,15.7]), sulfur (22.7 μm [15.0,30.4]) and total PM_2.5_ (14.7 μm [9.0,20.5]) but not silicon (5.2 μm [−9.8,20.1]), were associated with increased CIMT; in two-pollutant models, only the association with OC was robust to control for the other pollutants. Findings were generally consistent across the three exposure estimation approaches. None of the PM measures were positively associated with either the presence or extent of CAC. In sensitivity analyses, effect estimates for OC and silicon were particularly sensitive to control for metropolitan area.

**Conclusion:**

Employing commonly-used exposure estimation approaches, all of the PM_2.5_ components considered, except silicon, were associated with increased CIMT, with the evidence being strongest for OC; no component was associated with increased CAC. PM_2.5_ chemical components, or other features of the sources that produced them, may be important in determining the effect of PM exposure on atherosclerosis. These cross-sectional findings await confirmation in future work employing longitudinal outcome measures and using more sophisticated approaches to estimating exposure.

## Background

Epidemiological studies have linked elevated levels of fine particulate matter (PM_2.5_) air pollution to an increased risk of cardiovascular mortality and morbidity [1]. PM_2.5_ represents a heterogeneous mixture of particles generated by many sources. The U.S. National Research Council has emphasized the importance of identifying characteristics of PM that contribute to its toxicity [2]. However, there is little consistent evidence as to whether some chemical components or sources of PM_2.5_ are associated with greater risks. In cohort studies, Pope *et al.*[3] and Dockery *et al.*[4] both reported that long-term exposure to PM_2.5_ sulfate was associated with cardiopulmonary mortality. Ostro *et al.* also provided evidence that long-term exposures to PM_2.5_ and several PM_2.5_ components (EC, OC, sulfate, etc.) were associated with increased risks of all-cause and cardiopulmonary mortality [5].

Most cohort studies on air pollution and cardiovascular health have been limited to estimating effects on cardiovascular events such cardiovascular death or incidence of cardiovascular disease [4,6-13]. Recently, cardiovascular cohorts have employed subclinical measures of cardiovascular disease, such as carotid intima-media thickness (CIMT), coronary artery calcium (CAC), and the ankle-brachial index (ABI) [14-16] that are predictive of future clinical cardiovascular events [17].

The Multi-Ethnic Study of Atherosclerosis (MESA) is a cohort study in six U. S. metropolitan areas involving four racial-ethnic groups (non-Hispanic white, African American, Hispanic and Chinese) designed to assess the prevalence, correlates and progression of subclinical cardiovascular disease [18]. The Multi-Ethnic Study of Atherosclerosis and Air Pollution (MESA Air) is an ancillary study to MESA designed to investigate effects of individual-level exposures to ambient PM_2.5_ and traffic-associated pollutants on subclinical and clinical cardiovascular disease in the MESA cohort [19]. Individual-level exposures will be estimated incorporating cohort-specific pollutant monitoring, spatio-temporal modeling, and eventually participant time-activity information. One part of the Health Effect Institute’s National Particle Components Toxicity (NPACT) Initiative is an ancillary study to MESA Air designed to use cohort-specific PM_2.5_ speciated monitoring data to identify the role of chemical components of PM_2.5_. Here we report findings based on MESA, MESA Air and NPACT on the cross-sectional associations between two subclinical measures, CIMT and CAC, and exposure to both PM_2.5_ and PM_2.5_ chemical components. We employ more commonly-used approaches to estimating exposure than those that will ultimately be used with an eye to being able to assess the impact in this planned paper on the health findings of using more sophisticated exposure estimates.

## Methods

### Subjects and geocoding

The original MESA cohort consisted of 6,814 men and women aged 45–84 years who were free of clinical cardiovascular disease at the baseline examination in 2000–2002. Detailed MESA eligibility criteria are available online (http://www.mesa-nhlbi.org). Individuals were recruited from communities near six field centers: Baltimore, Maryland; Chicago, Illinois; Forsyth County (Winston-Salem), North Carolina; Los Angeles County, California; New York City, New York; and St. Paul, Minnesota. Details of the sampling plan have been previously reported [18]. The present analyses are based on health data collected at the baseline visit (July 2000-August 2002). The study was reviewed and approved by the Institutional Review Board of the University of Washington Human Subjects Division. Institutional review board approval was also granted at each study site and written informed consent was obtained from all participants.

Participants’ residential addresses at baseline were assigned geographic coordinates using ArcGIS 9.1 software (ESRI, Redlands, CA). Addresses were manually cleaned prior to geocoding, and matches with a geocoding score less than or equal to 80% were checked manually for accuracy. Of the 6,814 MESA cohort members at baseline, 6,256 consented to geocoding and were successfully geocoded.

### Outcome and risk factor data

Subclinical cardiovascular disease measures of CIMT and CAC were used as the primary endpoints. Trained technicians obtained images on supine study participants of the right common carotid artery using high resolution B-mode ultrasound (Logiq 700, 13 MHz; GE Medical Systems, Waukesha, Wisconsin). Images were obtained over a distance of 10 mm proximal to the common carotid bulb and transferred from each study center to Tufts Medical Center for measurement of CIMT. The mean far wall thickness of the right common carotid, retrospectively gated to end-diastole, was used for the analysis [20]. CAC was measured by chest computed tomography (CT) using a cardiac-gated electron beam computed tomography scanner (Imatron C-150; Imatron, San Francisco, California) at three field centers (Chicago, Los Angeles County, New York) and a retrospectively gated multi-detector row computed tomography system (Lightspeed, General Electric Medical Systems, Waukesha, Wisconsin; or Volume Zoom, Siemens, Erlanger, Germany) at the other three field centers (Baltimore, Winston-Salem, St. Paul) [21]. All scans used phantoms of known physical calcium concentration. Two CT scans were obtained for each participant. Scans were read centrally at the Harbor-UCLA Research and Education Institute in Torrance, California. The mean Agatston score [22] of the two scans was used for the analysis. The presence of CAC was defined as an Agatston score greater than zero.

Data on demographics, lifestyle characteristics, cardiovascular risk factors, medical history, and use of medications were obtained from detailed questionnaires.

### Air pollution exposure estimation

**Figure 1:**
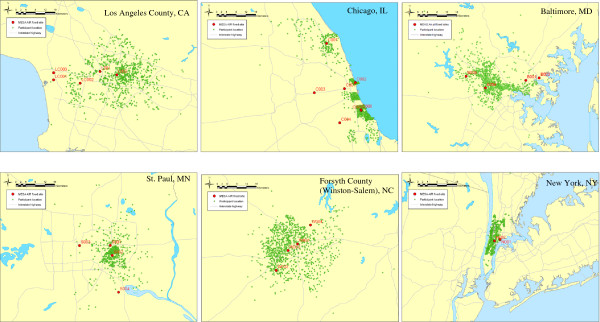
**Maps of the six MESA areas at the same scale showing MESA Air fixed monitoring site locations (numbers correspond to monitor ID [Table**2**]), jittered study participant locations and interstate highways.**

Details of the MESA Air monitoring site locations and protocol have been reported previously [23]. Figure 1 shows MESA Air fixed monitoring site locations in the six MESA areas. In general, one monitor was collocated with a U. S. Environmental Protection Agency Chemical Speciation Network (CSN) PM_2.5_ monitor, one near a roadway, and one in an area near a large concentration of study participant residences. Specifically, five monitors were sited in Los Angeles County, CA; three monitors in St. Paul, MN; four monitors in Forsyth County, NC; five monitors in Chicago, IL; four monitors in Baltimore, MD; and two monitors in New York City, NY. The location for these sites included libraries, schools, or other buildings that were in participant-dense areas underrepresented by the existing CSN. MESA Air and NPACT monitoring consisted of two-week samples of PM_2.5_ obtained on Teflon and quartz filters for every two weeks during the study period (see below). In addition to measurement of PM_2.5_ mass, Teflon filters underwent X-ray fluorescence (XRF) for trace elements and metals (Cooper Environmental Services, Portland, OR); EC and OC were measured on the quartz filters using the IMPROVE-A/thermal optical reflectance (TOR) method (Sunset Laboratory Inc., Tigard, OR). Monitoring at MESA Air sites began in July, 2005 and ended in August 2009; quartz filters were only deployed from April 2007 through August 2008. Data from the 50-week period from May 2, 2007 to April 16, 2008 was used for this analysis in order to obtain a near full year of data on all components. To deal with outliers, the mean for each monitoring site over that period was calculated as the 10 percent trimmed mean (i.e., the top and bottom 5 percent of data were excluded for the calculation of the mean). The following PM_2.5_ component concentrations were included in this analysis: elemental carbon [EC], organic carbon [OC], silicon and sulfur. EC and OC were selected as reflecting combustion sources, silicon as an indicator of crustal dust and sulfur as an indicator of sulfate, a secondary aerosol.

Three different approaches were used to estimate study participant exposure to PM_2.5_ components within each of the six areas corresponding to field centers: (1) the annual average concentration of the two-week measurements at the monitor nearest to each study participant’s residence at the baseline examination (“nearest monitor”); (2) inverse distance weighting (IDW) of all annual average monitor concentrations in each area relative to each subject’s residence; and (3) city-wide average concentrations based on all monitors within each area.

For all three approaches, participants within each area residing within 100 meters of either an A1 road (primary limited access or interstate highway) or an A2 road (primary US or state highway, without limited access), or within 50 meters of an A3 road (secondary state or county highway), were considered “near roadway” participants. Because PM_2.5_ and EC concentrations are strongly influenced by proximity to major roadways, for all approaches, near roadway participants were assigned the average PM_2.5_ and EC concentrations measured at that area’s MESA Air roadside monitor. The roadside monitors were not used in calculating the PM_2.5_ and EC exposures of participants not living close to an A1, A2 or A3 road. For OC, silicon and sulfur, roadside monitors were included in the calculations for all three of the exposure estimation methods.

To obtain information on temporal trends, PM_2.5_ component data were obtained from the Health Effects Institutes (HEI) Air Quality Database website (https://hei.aer.com/login.php) for 2002 and 2007. Because there were no CSN PM_2.5_ component data in St. Paul, Minneapolis data were used instead.

### Statistical analysis

Multiple linear regression was used to estimate the associations between PM_2.5_ measures and CIMT and CAC (among persons with Agatston scores greater than zero). Agatston scores were analyzed after log transformation. Relative risk regression was used to estimate the associations between PM_2.5_ measures and the presence of CAC (Agatston scores>0) [24]. All measures of association were expressed per inter-quartile range (IQR) of each concentration measure.

Covariates in our base model (Model 1) included age, gender, and race/ethnicity. Covariates included in our primary health model (Model 2) were selected based on the Framingham Risk Score [25]. In addition to Model 1 covariates, Model 2 included total cholesterol, HDL cholesterol, smoking status, hypertension, and lipid lowering medication. An extended set of covariates was included in Model 3 that added potential risk factors not included in the Framingham Risk Score: level of education, income, waist circumference, body surface area, body mass index (BMI) and squared BMI, diabetes, LDL cholesterol and triglycerides. In Model 4, metropolitan areas were added as indicator variables to our primary model (Model 2).

Race/ethnicity was categorized as white non-Hispanic of European ancestry, Chinese, African American, or Hispanic, as reported by the study participants. BMI, total cholesterol, HDL cholesterol, LDL cholesterol, triglycerides, waist circumference and body surface area were included as continuous variables. Cigarette smoking status was categorized as never, former or current smoker. Annual family income was categorized into 5 categories. Education was classified as: high school not completed, high school completed, some college but no degree, or completed bachelor’s degree or more. Current use of lipid-lowering medications was classified as either some or none. Diabetes was categorized as not diabetic, impaired fasting glucose (defined fasting glucose =5.5-6.9mmol/L (100-125mg/dL)), untreated diabetes and treated diabetes. Diabetes was defined as fasting glucose of ≥ 7.0mmol/L (≥ 126 mg/dL) or use of hypoglycemic medication. Hypertension was defined as systolic blood pressure ≥140 mmHg, diastolic blood pressure ≥ 90 mmHg or taking antihypertensive medications.

Models using the nearest monitor concentration estimates and the primary set of health covariates (Model 2) were considered as our primary analytic models. Sensitivity analysis included comparing findings across the four sets of covariate models and the three alternative exposure measures, as well as assessing estimates from selected two-pollutant models and models for CIMT that controlled for ultrasound sonographer.

All data analyses were performed using R 2.12.2 [26].

## Results

### Subject characteristics

Mean age of the study participants was 62 years, 47.5 percent were male, and 39.1 percent were non-Hispanic white, 11.7 percent Chinese, 27.4 percent African American, and 21.7 percent Hispanic. Additional characteristics of the study sample are shown in Table 1. The prevalence of current smoking was low (12.7%), and approximately half of the cohort reported never having smoked.

**Table 1:** Participant characteristics (total N=6,256) at the baseline examination 2000-2002

	**N**	**%**		**N/mean**	**%/SD**
Gender			Diabetes		
Male	2974	47.5	Normal	4635	74.1
Female	3282	52.5	IFG*	855	13.7
Age(years)			Treated diabetes	157	2.5
45-54	1828	29.2	Untreated diabetes	589	9.4
55-64	1755	28.1	Missing	20	0.3
65-74	1838	29.4	Education		
75-84	835	13.3	Less than high school graduate	1057	16.9
Race-ethnicity			High school graduate	1135	18.1
White	2449	39.1	Some college	1776	28.4
Chinese	735	11.7	College graduate or higher	2269	36.3
Black	1714	27.4	Missing	19	0.3
Hispanic	1358	21.7	Lipid lowering medication		
Income ($/year)			No	5238	83.7
<12,000	655	10.5	Yes	1015	16.2
12,000-24,999	1161	18.6	Missing	3	0.05
25,000-49,999	1748	27.9	Hypertension		
50,000-74,999	1048	16.8	No	3504	56.0
≥75,000	1410	22.5	Yes	2752	44.0
missing	234	3.7	MESA area		
Cigarette smoking			Forsyth County (Winston-Salem)	999	16.0
Never	3145	50.3	New York	1021	16.3
Former	2299	36.7	Baltimore	975	15.6
Current	794	12.7	St. Paul	982	15.7
Missing	18	0.3	Chicago	1088	17.4
BMI*			Los Angeles County	1191	19.0
<23	1796	28.7	Total choleterol (mg/dl)	194.1	35.4
23-27.5	2459	39.3	HDL choleterol (mg/dl)	50.9	14.7
27.6-40	1777	28.4	LDL choleterol (mg/dl)	117.2	31.2
>40	224	3.6	Triglycerides (mg/dl)	131.0	86.6
			Waist circumference(cm)	98.0	14.3
			Body surface area	1.9	0.2

* BMI=body mass index; IFG=impaired fasting glucose.

Median CIMT was 0.84 mm (IQR 0.23 mm) in the 6,183 study participants with CIMT. Forty nine percent of the 6,256 participants with a CAC measurement had an Agatston score greater than 0; among those, the median score was 86.0 Agatston units (IQR 270.5).

Two hundred and eight subjects (3.3%) lived within 100 meters of an A1 road, 243 (3.9%) with 100 meters of an A2 road, and 1,459 (23.3%) within 50 meters of an A3 road. A total of 1,774 subjects (28.4%) were therefore classified as living close to a major roadway. Using the criteria for living close to a major roadway, the following in each area lived close to a major roadway: 18.5% in Forsyth County (Winston-Salem), 59.5% in New York, 20.8% in Baltimore, 22.8% in St. Paul, 31.2% in Chicago, and 18.1% in Los Angeles County. Among those not living close to a major roadway, median distance to the nearest MESA Air monitor was 4.1 km (IQR 4.3); 90.3% of the participants resided within 10 km of a MESA Air monitor.

### PM_2.5_ and PM_2.5_ component concentrations

**Figure 2:**
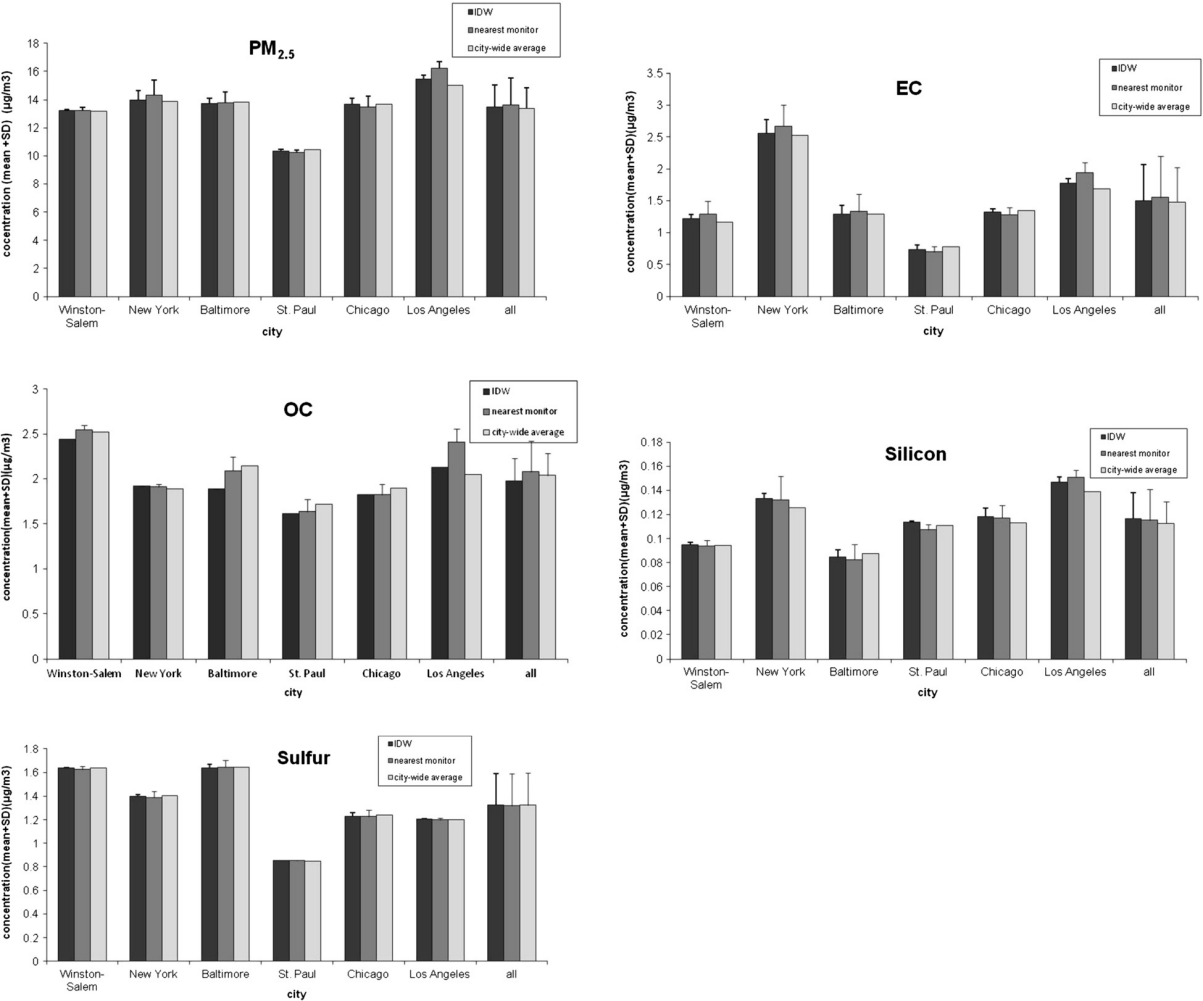
**Annual average PM**_**2.5 **_**and PM**_**2.5 **_**component concentrations (mean and standard deviation bar) by metropolitan area and exposure estimation approach.** PM_2.5_, particulate matter less than or equal to 2.5 μm in aerodynamic diameter; EC, elemental carbon; OC, organic carbon; IDW = inverse distance weighting.

Table 2 shows PM_2.5_ and PM_2.5_ component annual average concentrations (μg/m^3^) by metropolitan area and monitor site. In general, PM_2.5_ and EC concentrations were highest at the roadside monitors. Table 3 shows median (IQR) study subject PM_2.5_ and component concentrations for the three exposure metrics. Mean PM_2.5_ concentrations for the six study areas based on nearest monitor ranged from 16.2 μg/m^3^ (Los Angeles County) to 10.3 μg/m^3^ (St. Paul); EC ranged from 2.7 μg/m^3^ (New York City) to 0.7 μg/m^3^ (St. Paul); OC ranged from 2.5 μg/m^3^ (Winston-Salem) to 1.6 μg/m^3^ (St. Paul); silicon ranged 0.15 μg/m^3^ (Los Angeles County) to 0.08 μg/m^3^ (Baltimore); and sulfur ranged from 1.7 μg/m^3^ (Baltimore) to 0.9 μg/m^3^ (St. Paul) (Figure 2). Strong correlations (Pearson’s correlation coefficient greater than 0.70) were present for PM_2.5_ and EC (0.74 for nearest monitor), and OC and sulfur (0.80 for city-wide average). Figure 2 also shows standard deviations of the means of the three exposure predictions for each PM_2.5_ component by city and for all cities combined. The within-city variability is markedly less than the variability for all cities combined.

**Table 2:** **PM**_**2.5 **_**and PM**_**2.5 **_**component annual average concentrations (μg/m**^**3**^**) by area and MESA Air monitor**

**City**	Monitor*	**Type**	**PM**_2.5_**	EC**	OC**	**Silicon**	**Sulfur**
LA County	L001	non-roadside	16.3	2.0	2.6	0.15	1.18
	L002	roadside	16.8	2.1	2.4	0.16	1.22
	LC001	non-roadside	13.4	1.5	1.6	0.13	1.20
	LC002	non-roadside	15.3	1.6	2.2	0.14	1.23
	LC003	roadside	13.3	1.4	1.4	0.12	1.18
Chicago	C001	non-roadside	12.2	1.2	1.7	0.10	1.13
	C002	non-roadside	13.7	1.3	1.8	0.13	1.19
	C004	non-roadside	14.6	1.6	2.0	0.10	1.31
	C006	non-roadside	13.8	1.3	1.8	0.12	1.26
	C007	roadside	15.5	1.7	2.1	0.12	1.30
Baltimore	B001	roadside	15.6	2.1	2.4	0.11	1.69
	B003	non-roadside	14.7	1.4	2.3	0.09	1.69
	B004	non-roadside	13.9	1.4	2.1	0.08	1.67
	B005	non-roadside	12.7	1.0	1.9	0.07	1.53
St. Paul	S001	roadside	11.0	1.1	1.9	0.12	0.87
	S002	non-roadside	10.2	0.7	1.6	0.11	0.85
	S003	non-roadside	10.5	0.8	1.7	0.11	0.83
NY	N001	non-roadside	13.2	2.3	1.9	0.11	1.46
	N002	roadside	15.4	3.0	1.9	0.15	1.36
Forsyth County	W001	non-roadside	13.2	1.5	2.6	0.09	1.62
(Winston-Salem)	W002	non-roadside	13.2	1.1	2.5	0.10	1.59
	W003	roadside	13.9	1.2	2.6	0.10	1.66
	W004	non-roadside	13.0	1.0	2.4	0.09	1.67

* May 2, 2007 – Apr 16, 2008, see Figure 1.

**PM_2.5_, particulate matter less than or equal to 2.5 μm in aerodynamic diameter; EC, elemental carbon; OC, organic carbon.

**Table 3:** **Distribution of PM**_**2.5 **_**and PM**_**2.5 **_**component concentrations (μg/m**^**3**^**) by three estimation approaches**

**Approach**	**PM**_**2.5**_		**EC**		**OC**		**Silicon**		**Sulfur**	
	**Median**	**IQR**	**Median**	**IQR**	**Median**	**IQR**	**Median**	**IQR**	**Median**	**IQR**
Nearest monitor	13.66	2.340	1.36	0.825	1.93	0.62	0.11	0.074	1.30	0.409
Inverse-distance weighting (IDW)	13.69	1.314	1.32	0.570	1.92	0.31	0.12	0.039	1.26	0.425
City-wide average	13.57	1.143	1.32	0.509	2.05	0.26	0.11	0.031	1.24	0.435

*PM_2.5_, particulate matter less than or equal to 2.5 μm in aerodynamic diameter; EC, elemental carbon; OC, organic carbon.

**Figure 3:**
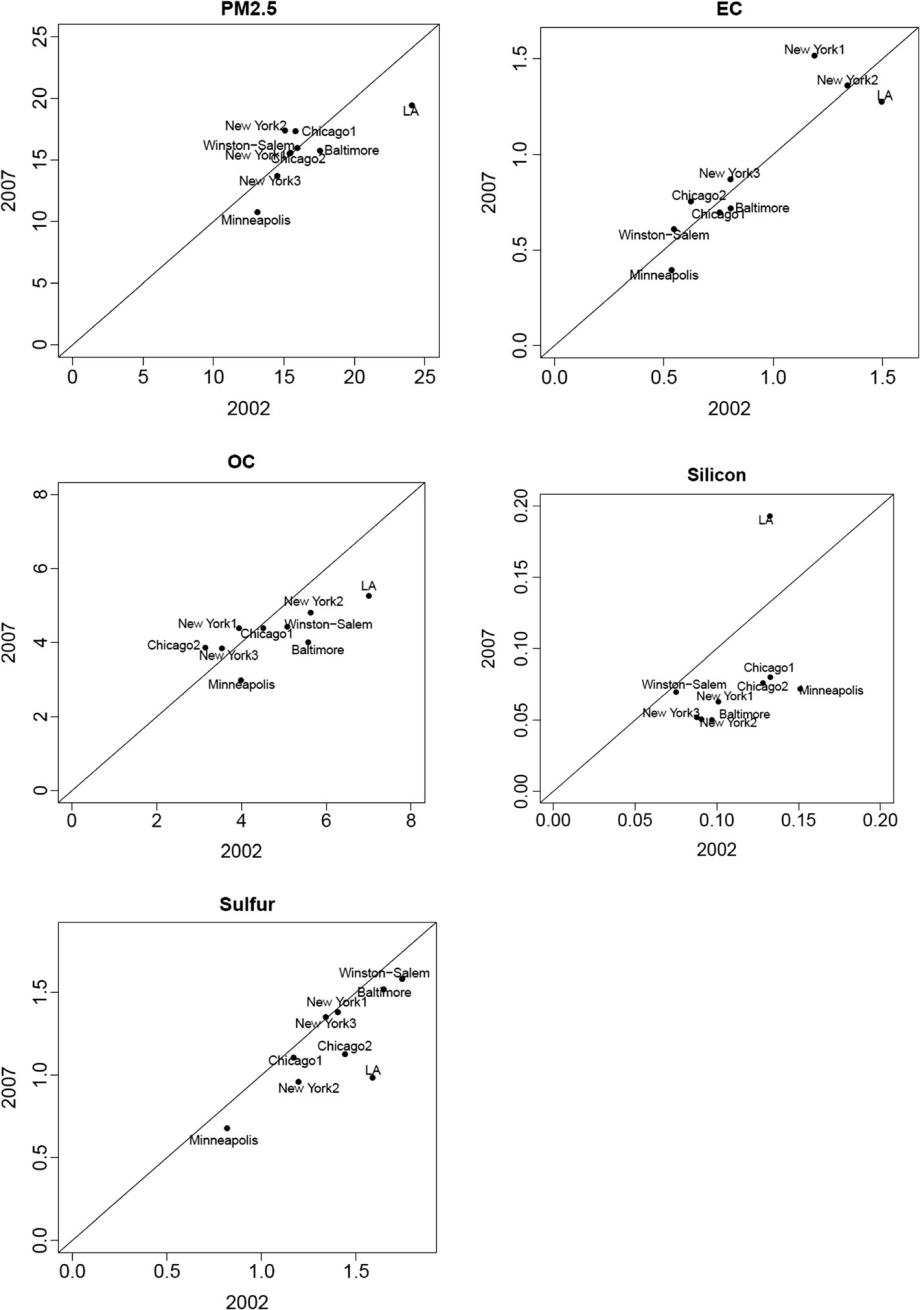
**Correspondence of mean PM**_**2.5 **_**and PM**_**2.5 **_**component concentrations in 2002 and 2007 from CSN monitoring sites in the MESA areas.** PM_2.5_ = particulate matter less than or equal to 2.5 μm in aerodynamic diameter; EC = elemental carbon; OC = organic carbon. Correlation coefficients for 2002 and 2007 values: PM_2.5_ (0.74), EC (0.91), OC (0.72), silicon (0.46), sulfur (0.79).

In order to assess stability of PM_2.5_ and PM_2.5_ component concentrations over time, we used data from the CSN monitoring network. Figure 3 shows the correspondence between mean PM_2.5_ and PM_2.5_ component concentrations from the CSN for 2002 and 2007 in the MESA areas with available CSN data. There was generally good correspondence between concentrations over that five-year span, except for silicon, which showed a decrease in four of the six MESA areas.

### CIMT

**Figure 4:**
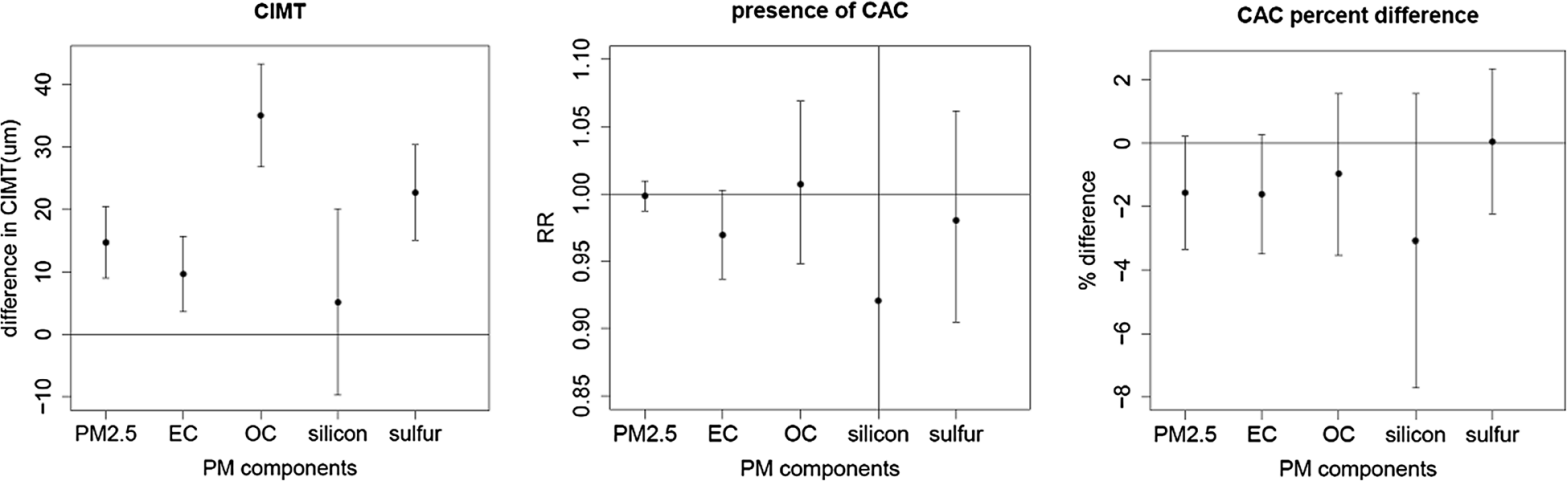
**Estimated effects of PM**_**2.5 **_**and PM**_**2.5 **_**components (per IQR) on CIMT (μm), presence of CAC, and amount of CAC based on nearest monitor exposure estimates and the primary covariate model (model 2, see Methods).** PM_2.5_ = particulate matter less than or equal to 2.5 μm in aerodynamic diameter; EC = elemental carbon; OC = organic carbon.

Table 4 shows estimates of effects of PM_2.5_ and PM_2.5_ components on CIMT by the several exposure estimation methods and analysis models. Figure 4 shows estimated effects on CIMT based on our primary exposure approach (nearest monitor) and Model 2. Increases in predicted PM_2.5_, OC, EC and sulfur, but not silicon, were associated with increased CIMT; CIMT increases per IQR concentration increases were 14.7 μm (95% CI [9.0,20.5]), 35.1 μm (26.8,43.3), 9.6 μm (3.6,15.7), 22.7 μm (15.0,30.4) and 5.2 μm (−9.8,20.1) for PM_2.5_, OC, EC, sulfur and silicon, respectively. The size of the effect estimates for OC and sulfur was higher than that for EC.

**Table 4:** CIMT* difference (μm) for pollutant IQR increases by analysis model and exposure estimation approach

	**PM**_2.5_*	EC*	OC*	**Silicon**	**Sulfur**
	**CIMT difference (95% CI)**	**CIMT difference (95% CI)**	**CIMT difference (95% CI)**	**CIMT difference (95% CI)**	**CIMT difference (95% CI)**
Model 1**					
Nearest Monitor	13.7 (8.0,19.5)	8.2 (2.2,14.2)	36.5 (28.3,44.7)	3.1 (−11.9,18.0)	22.6 (14.9,30.2)
IDW	8.8 (4.8,12.7)	4.8 (0.0,9.5)	25.4 (19.9,30.8)	−0.6 (−10.2,9.0)	23.8 (15.8,31.8)
City-Wide Average	7.6 (3.7,11.3)	2.6 (−1.9,7.1)	21.3 (16.4,26.2)	−0.2 (−9.9,9.5)	22.9 (14.8,31.0)
Model 2**					
Nearest Monitor	14.7 (9.0,20.5)	9.6 (3.6,15.7)	35.1 (26.8,43.3)	5.2 (−9.8,20.1)	22.7 (15.0,30.4)
IDW	9.6 (5.7,13.5)	6.0 (1.3,10.8)	24.9 (19.4,30.3)	1.3 (−8.3,10.9)	24.0 (16.0,32.0)
City-Wide Average	8.5 (4.7,12.3)	3.9 (−0.5,8.4)	20.6 (15.7,25.5)	1.8 (−7.9,11.4)	23.3 (15.2,31.4)
Model 3**					
Nearest Monitor	15.8 (9.9,21.7)	10.8 (4.6,16.9)	36.5 (28.0,44.9)	7.3 (−8.0,22.5)	23.9 (16.0,31.7)
IDW	10.6 (6.5,14.6)	7.2 (2.3,12.0)	26.9 (21.2,32.6)	2.4 (−7.4,12.2)	25.4 (17.2,33.5)
City-Wide Average	9.6 (5.7,13.5)	5.0 (0.4,9.6)	21.8 (16.7,26.9)	2.8 (−7.1,12.7)	24.8 (16.5,33.1)
Model 4**					
Nearest Monitor	5.9 (−10.3,22.0)	6.3 (−11.4,23.8)	−2.3 (−26.2,21.5)	−10.1 (−41.0,20.6)	27.4 (−19.3,73.8)
IDW	6.0 (−9.8,21.8)	12.1 (−11.0,35.0)	***	−8.5 (−47.2,30.0)	***

*PM_2.5_, particulate matter less than or equal to 2.5 μm in aerodynamic diameter; EC, elemental carbon; OC, organic carbon; CIMT, carotid intima-media thickness.

**Model 1: covariates include age, gender, race-ethnicity.

Model 2: Model 1 + total cholesterol, HDL cholesterol, smoking status, hypertension, lipid-lowering medication.

Model 3: Model 2 + education, income, waist circumference, body surface area, BMI, BMI^2^, diabetes, LDL, triglycerides.

Model 4: Model 2 + metropolitan area.

***unstable estimate.

In sensitivity analyses, findings were generally consistent across the three exposure estimation approaches and were largely unchanged when controlling for more covariates in the extended model (Model 3). In addition to controlling for lipid-lowering medications in our primary model, we carried out an analysis restricted to those who reported never having been on statin medications (n= 4,754); findings in this subgroup were essentially identical to those in the larger group (results not shown). Effects of adding variables for each metropolitan area to the model (Model 4), effectively removing between-area effects and allowing assessment of only within-area effects, were also examined. Metropolitan area variables could not be added to models in which city-wide average was used as the exposure metric. Several findings were sensitive to control for area. For our primary exposure method and model, none of associations of PM_2.5_ and PM_2.5_ components with CIMT were significant when metropolitan areas were included as covariates in model 2 (Model 4) (Table 4), although the size of the effect estimates for EC and sulfur remained essentially unchanged, and the effect of PM_2.5_ was only moderately reduced. We also included ultrasound sonographer as an indicator variable in place of metropolitan area in the CIMT models for sonographers who performed at least 10 studies. Since sonographers were unique to study site, this effectively also controlled for study area. Results with sonographer in the CIMT models were essentially no different from those controlling for metropolitan area (results not shown).

For CIMT, estimates from two-pollutant models were examined using nearest monitor and the primary analysis model that included each pair of the PM_2.5_ components. Only the association of CIMT with OC was not sensitive to inclusion in the model of the other components or total PM_2.5_ (results not shown).

### CAC

Table 5 shows estimated effects of PM_2.5_ and PM_2.5_ components on presence of CAC by the several estimation methods and models. Figure 4 shows estimated effects on presence of CAC based on our primary exposure approach (nearest monitor) and Model 2. For this model, there were no statistically significant associations between presence of CAC and PM_2.5_ or PM_2.5_ components. In sensitivity analyses, using IDW or city-wide average, presence of CAC was negatively associated with EC in Model 2 and in Model 3 with the extended set of covariates. With adjustment for metropolitan region (Model 4), EC was no longer negatively associated with presence of CAC.

**Table 5:** CAC* relative risk (RR) for pollutant IQR increases by analysis model and exposure estimation approach

	**PM**_2.5_*	EC*	OC*	**Silicon**	**Sulfur**
	**RR (95% CI)**	**RR (95% CI)**	**RR (95% CI)**	**RR (95% CI)**	**RR (95% CI)**
Model 1**					
Nearest Monitor	1.00 (0.99,1.01)	0.96 (0.93,0.99)	1.03 (0.96,1.09)	0.84 (0.33,2.14)	0.98 (0.91,1.07)
IDW	0.99 (0.98,1.01)	0.95 (0.91,0.99)	0.98 (0.90,1.06)	0.49 (0.16,1.53)	0.98 (0.90,1.06)
City-Wide Average	0.99 (0.98,1.00)	0.94 (0.90,0.98)	1.01 (0.09,1.55)	0.37 (0.09,1.55)	0.97 (0.89,1.05)
Model 2**					
Nearest Monitor	1.00 (0.99,1.01)	0.97 (0.94,1.00)	1.01 (0.95,1.07)	0.92 (0.37,2.32)	0.98 (0.90,1.06)
IDW	1.00 (0.98,1.01)	0.96 (0.92,1.00)	0.96 (0.89,1.04)	0.64 (0.21,1.96)	0.97 (0.90,1.05)
City-Wide Average	0.99 (0.98,1.01)	0.95 (0.91,0.99)	0.99 (0.91,1.08)	0.51 (0.12,2.09)	0.97 (0.89,1.05)
Model 3**					
Nearest Monitor	1.00 (0.99,1.01)	0.97 (0.94,1.01)	1.03 (0.97,1.09)	1.02 (0.39,2.64)	0.99 (0.91,1.07)
IDW	1.00 (0.99,1.01)	0.96 (0.92,1.00)	0.99 (0.91,1.07)	0.70 (0.22,2.22)	0.98 (0.90,1.06)
City-Wide Average	1.00 (0.98,1.01)	0.96 (0.91,0.99)	1.01 (0.93,1.10)	0.57 (0.13,2.44)	0.97 (0.90,1.06)
Model 4**					
Nearest Monitor	1.01 (0.98,1.05)	1.04 (0.94,1.15)	1.08 (0.91,1.28)	***	1.35 (0.79,2.30)
IDW	1.02 (0.97,1.09)	1.11 (0.91,1.34)	***	***	2.04 (0.62,6.66)

*PM_2.5_, particulate matter less than or equal to 2.5 μm in aerodynamic diameter; EC, elemental carbon; OC, organic carbon; CAC, coronary artery calcification.

**Model 1: covariates include age, gender, race-ethnicity.

Model 2: Model 1 + total cholesterol, HDL cholesterol, smoking status, hypertension, lipid-lowering medication.

Model 3: Model 2 + education, income, waist circumference, body surface area, BMI, BMI^2^, diabetes, LDL, triglycerides.

Model 4: Model 2 + metropolitan area.

***unstable estimate.

Table 6 shows estimated effects of PM_2.5_ and PM_2.5_ components on log-transformed CAC (in those with detectable calcium) by estimation method and models. Figure 4 shows estimated effects on CAC in those with measurable CAC based on our primary exposure and analysis model. For the primary exposure and analysis model, no significant positive association of any PM measure and amount of CAC was observed. In sensitivity analyses, silicon was associated with amount of CAC using city-wide average and IDW, but in the negative direction; this negative association was no longer present after adjustment for city region (Model 4).

**Table 6:** Percentage change in CAC* for pollutant IQR increases by analysis model and exposure estimation approach

	**PM**_2.5_*	EC*	OC*	**Silicon**	**Sulfur**
	**% Change (95% CI)**	**% Change (95% CI)**	**% Change (95% CI)**	**% Change (95% CI)**	**% Change (95% CI)**
Model 1**					
Nearest Monitor	−1.52 (−3.31,0.26)	−1.65 (−3.54,0.24)	−0.59 (−3.14,1.96)	−3.96 (−8.62,0.70)	0.61 (−1.67,2.89)
IDW	−1.00 (−2.21,0.21)	−1.26 (−2.76,0.24)	−0.61 (−2.29,1.08)	−3.14 (−6.09,-0.19)	0.70 (−1.68,3.08)
City-Wide Average	−0.85 (−2.02,0.32)	−1.11 (−2.52,0.30)	0.19 (−1.32,1.70)	−3.41 (−6.36,-0.45)	0.66 (−1.76,3.07)
Model 2**					
Nearest Monitor	−1.56 (−3.34,0.21)	−1.61 (−3.49,0.27)	−0.98 (−3.52,1.57)	−3.08 (−7.73,1.58)	0.05 (−2.22,2.33)
IDW	−1.03 (−2.23,0.17)	−1.22 (−2.71,0.28)	−0.86 (−2.54,0.83)	−2.52 (−5.47,0.43)	0.12 (−2.25,2.50)
City-Wide Average	−0.63 (−1.84,0.58)	−0.83 (−2.28,0.63)	0.13 (−1.44,1.70)	−3.66 (−6.71,-0.61)	1.13 (−1.34,3.60)
Model 3**					
Nearest Monitor	−1.37 (−3.22,0.48)	−1.43 (−3.37,0.52)	−0.86 (−3.51,1.78)	−3.35 (−8.18,1.47)	0.58 (−1.76,2.92)
IDW	−0.89 (−2.14,0.37)	−1.06 (−2.61,0.49)	−0.90 (−2.67,0.86)	−3.07 (−6.13,0.00)	0.67 (−1.77,3.12
City-Wide Average	−0.72 (−1.94,0.49)	−0.90 (−2.36,0.57)	−0.05 (−1.63,1.53)	−3.21 (−6.27,-0.14)	0.64 (−1.85,3.12)
Model 4**					
Nearest Monitor	−3.10 (−8.08,1.89)	−3.40 (−8.76,1.97)	−1.05 (−8.03,5.93)	4.13 (−5.43,13.68)	−2.32 (−16.91,12.27)
IDW	−3.78 (−8.75,1.19)	−5.42 (−12.52,1.69)	***	3.24 (−8.59,15.07)	8.51 (−24.75,41.77)

*PM_2.5_, particulate matter less than or equal to 2.5 μm in aerodynamic diameter; EC, elemental carbon; OC, organic carbon; CAC, coronary artery calcification.

**Model 1: covariates include age, gender, race-ethnicity.

Model 2: Model 1 + total cholesterol, HDL cholesterol, smoking status, hypertension, lipid-lowering medication.

Model 3: Model 2 + education, income, waist circumference, body surface area, BMI, BMI^2^, diabetes, LDL, triglycerides.

Model 4: Model 2 + metropolitan area.

***unstable estimate.

## Discussion

The MESA Air and NPACT air monitoring campaign employed cohort-oriented fixed site monitors in which 2-week measurements of total PM_2.5_ and PM_2.5_ components were obtained. Using our primary approach to estimating exposure (nearest monitor) and our primary health effects model, there were cross-sectional associations of CIMT with OC, EC, and sulfur, as well as with total PM_2.5_, but not with silicon. The strongest associations were for OC and sulfur. The associations were reasonably robust to exposure estimation method (nearest monitor, IDW and city-wide mean) and to adjustment for additional potential risk factors and other PM_2.5_ components, but not to control for metropolitan area. These associations with CIMT, especially that for OC, were therefore primarily related to exposure differences between metropolitan areas.

Neither presence of CAC nor extent of CAC was positively associated with PM_2.5_, OC, or any other PM_2.5_ components evaluated. EC and silicon in some models were associated with CAC, but in the negative direction; these associations were sensitive to control for metropolitan area. While CIMT and CAC are highly correlated, they independently predict future cardiovascular events [17], suggesting that each provides somewhat different information on atherosclerosis and cardiovascular disease risk. CAC is a measure of plaque in the coronary arterial bed while CIMT can be regarded more as a continuous measure of generalized atherosclerosis. Our findings of associations with CIMT but not with CAC are consistent with earlier findings on PM in the MESA cohort [15] and may indicate differential pollutant effects on different vascular beds.

CIMT has been associated cross-sectionally with ambient PM_2.5_ concentrations estimated using regulatory monitoring data and different approaches to estimating within-urban concentrations. Künzli *et al.* reported an association in Los Angeles between CIMT and PM_2.5_ estimated using kriging, with exposure assigned at the zip code level [14]. Diez Roux *et al.* reported an association between 20-year average PM_2.5_ concentration and CIMT in this same MESA cohort, with PM_2.5_ estimated using a spatio-temporal model to predict PM_2.5_ concentrations at each participant’s residence [15]. In this study, as in the current study, there was no association between either presence or extent of CAC and 20-year average PM_2.5_[15]. Hoffmann *et al.,* however, reported an association in the Heinz Nixdorf Recall Study cohort in Germany between PM_2.5_ estimated from a dispersion model and the amount of CAC in the subset of study subjects not working full-time [27]. Unlike our study, the primary focus in these prior studies was on PM_2.5_ total mass rather than PM_2.5_ chemical composition, and regulatory monitoring data were employed in estimating PM_2.5_ concentrations. Analyses involving longitudinal measures of subclinical outcomes and PM_2.5_ and PM_2.5_ components will help to assess the validity of our and others’ cross-sectional findings.

The cardiovascular effects of long-term exposure to several PM_2.5_ components have been examined in only one other study. Using the California Teachers Study (CTS) cohort, a prospective cohort of active and former female public school professionals, Ostro *et al.*, in corrected analyses, reported an association of PM_2.5_ total mass with cardiopulmonary and ischemic heart disease (IHD) mortality; associations of IHD mortality with several PM_2.5_ components, including OC, EC, sulfate and silicon, were observed [5]. Here we evaluated the associations between long-term concentrations of both PM_2.5_ total mass and selected PM_2.5_ components (EC, OC, silicon, and sulfur) and measures of subclinical atherosclerosis (CIMT and CAC). Of the PM components, the strongest and most consistent associations with CIMT were observed for OC and sulfur; only the association for OC was robust to control for the other components.

The choice of the four PM components was influenced by *a priori* notions that these components reflect different important sources, and together make up the majority of the PM_2.5_ mass. The OC carbon fraction reflects direct emissions from fossil fuel and biomass combustion and biogenic sources, as well as contributions from secondary atmospheric reactions [28,29]. In addition to the long-term exposure associations, short-term exposure effects of OC have also been reported in several time series studies. For example, short-term exposure effects on daily cardiovascular mortality were observed in six California counties and in Phoenix, Arizona [30,31]. Metzger *et al.* reported an association between OC and emergency department visits for cardiovascular disease in Atlanta, Georgia [32]. Modification of short-term PM_2.5_ cardiovascular effects by long-term concentrations of OC has also been reported [33], although this has not been a consistent observation [34,35].

Sulfur, used as a marker of sulfate, was also associated with CIMT. Sulfate is a secondary aerosol that is formed through photochemical reactions involving sulfur-based compounds, notably sulfur dioxide. Associations of sulfate with cardiopulmonary disease mortality were observed in the ACS study, the Six-Cities study and in the CTS cohort [5]. The sulfate component modified the short-term effect of PM_2.5_ in one mortality time series study [34]. Also, short-term effects of sulfate have been reported in some studies [36]. It is not clear whether the observed associations with sulfate indicate direct effects of sulfate, or whether these reflect effects of components in the secondary pollutant mix that includes sulfate.

The EC carbon fraction is typically used to reflect diesel emissions and other combustion processes such as wood burning [37,38]. We found some associations of EC with CIMT, but these were sensitive to inclusion of any one of the other PM components in the health model (results not shown). EC was negatively associated with presence of CAC, but not after controlling for metropolitan area. Long-term EC was associated with ischemic heart disease mortality in the CTS [5] and was reported to modify short-term PM_2.5_ effects in one study [35], but not in others [33,34]. Also, short-term EC exposure effects have been reported in time series studies [36].

We found little evidence of an association of silicon with CIMT. In contrast, silicon was, along with several PM_2.5_ components, associated with cardiopulmonary mortality in the CTS cohort [5]. Silicon is a crustal element that is a large component of soil and resuspended road dust [39]. It may therefore reflect constituents found in road dust, including combustion-based material, brake dust, tire debris, and semivolatile compounds. It may also serve as a general marker for proximity to traffic. Short-term exposure to silicon has been associated with cardiovascular effects in a few time-series studies in Arizona and California [2,30,31,40,41]. In contrast, in a study of six eastern and Midwestern cities in the U.S., no association between mortality and daily exposure to silicon was seen [42]. Long-term concentrations of silicon did not modify short-term PM_2.5_ effects in one study [35]. Silicon PM was associated with exacerbation of myocardial ischemia in a dog model of coronary artery disease [39]. While relatively limited exposure contrasts for silicon may have hampered our ability to detect associations with silicon, previously reported findings on silicon effects on cardiovascular outcomes have not been consistent.

All of our measures of PM exposure were based only on the MESA Air fixed ambient air monitors (Figure 1), as we were not able to integrate CSN data into our exposure estimates due to the well-documented poor comparability of monitoring methods, notably for OC and EC, during the monitoring period [43]. Three approaches were employed in assigning exposure to PM_2.5_ total mass and PM_2.5_ components. The nearest monitor and the IDW approaches assigned exposure based on the participant’s address, as has been done in a few other studies of long-term PM exposure effects on cardiovascular diseases [12,14]. The nearest monitor is assumed to provide a more valid estimate of exposure than city-wide mean, although it is not clear whether it is superior to the IDW approach. Both nearest monitor and IDW estimates attempt to capture some within-city variability in exposure, as opposed to the city-wide mean. We chose, somewhat arbitrarily, the nearest monitor approach as our primary exposure estimation approach, using the IDW and city-wide mean approaches in sensitivity analyses. Our findings were not highly sensitive to the approach to estimating exposure. Future analyses of PM_2.5_ component effects in the MESA cohort will take advantage of more sophisticated spatio-temporal modeling of pollutants, which will allow for assessment of the impact on the health findings of using these more sophisticated exposure estimates.

A limitation of all of our approaches to estimating exposure is that we only estimate outdoor residential concentrations rather than concentrations to which people are actually exposed. While outdoor concentrations have been shown to be reasonable proxies for indoor concentrations and for personal exposure to particles of outdoor origin [44,45], estimates of outdoor concentrations necessarily mismeasure exposures that are influenced by time-activity patterns that take people away from home, such as work and travel to work. Time-activity studies, however, show that people spend most of their time in or around home [46], justifying the common practice of basing exposure estimates on place of residence. In MESA Air, time-activity data from the entire MESA cohort confirmed that study participants spent most of their time in their homes; the elderly or Chinese participants spent relatively more time in their homes [47]. Future analyses will assess whether our estimates of health effect are modified by incorporation of data collected on time-activity patterns and infiltration of particles indoors.

Adding to the complexity of PM exposure measurement error is the likely differential measurement error across the different PM_2.5_ components. It is expected that sulfate, with relatively homogeneous concentrations within a metropolitan area, would exhibit less measurement error than OC, for example, whose concentrations vary within an urban area [48]. To the extent that increased exposure measurement error biases effect estimates toward the null [35], it is possible that the association with sulfate is underestimated to a lesser extent than that with OC. In spite of that, we observed that the effect per IQR was higher for OC than any of our PM_2.5_ components, suggesting that the true association for OC may have been even larger.

Exposure misclassfication could also result from estimating exposure for a time period that is not relevant to the exposure responsible for the observed effect. Exposure was assigned based on one year of monitoring from 2007 to 2008, whereas our endpoint measurements were obtained during the period 2000 to 2002. To address the issue of PM component concentration stability over time, we examined CSN PM_2.5_ component data monitoring sites in the six MESA areas for the years 2002 and 2007. There was generally good correlation over that 5-year span (Figure 3). It is therefore reasonable to assume that concentrations in 2000–2002, while likely higher than those in more recent years, were nevertheless highly correlated with them. In the MESA cohort, PM_2.5_ concentrations were highly correlated over a 20-year period [15], as they were in the American Cancer Society study [49].

Although we included a reasonably comprehensive list of potential individual-level confounder variables in our health effect analyses, it is possible that uncontrolled confounding from unmeasured confounders associated with metropolitan area is present. This motivated control for metropolitan area in Model 4. Our findings were variably sensitive to control for metropolitan area. For example, when study area was added to models using our primary estimation approach (nearest monitor), there was no longer an association of some of the PM_2.5_ components, especially OC, with CIMT. Because much of the variability in exposure was due to variability between areas, control for metropolitan area substantially reduced exposure variability, which limits our power to detect associations. While we put most interpretive weight on models that did not control for metropolitan area, it may have been preferable to place more weight on findings from models with control for metropolitan area if it had been possible to accomplish that without dramatically reducing variability in exposure.

Strengths of this study include the wealth of detailed information on cardiovascular risk factors, the standardized assessment of outcomes, the attempt to incorporate some features of within-city variability in our exposure estimates based on PM_2.5_ species air monitoring carried out specifically on the MESA cohort, and the assessment of sensitivity of findings to employing three commonly-used approaches for estimating exposure. Future work will employ more sophisticated methods for estimating individual-level exposure to PM components that incorporate land use regression modeling and geostatistical methods, as well as time-activity data. Effects on longitudinal change in CIMT and CAC, in addition to the cross-sectional effects described in this report, will also be assessed when those data are available.

## Conclusion

In summary, this is the first study to assess the effect of PM_2.5_ chemical components on subclinical measures of cardiovascular disease. In this multi-ethnic cohort using the nearest monitor approach to estimating exposure, of the selected PM_2.5_ components, we found the strongest evidence for cross-sectional associations of OC and sulfur with CIMT. More sophisticated exposure estimation is planned in the MESA cohort, as well as analyses utilizing longitudinal outcome measures, either of which could enhance the validity of future health effect estimates. Evidence such as reported here on the differential health effects of individual PM components should allow for more focused and effective ambient air quality standards aimed at protecting public health.

## Abbreviations

CIMT: Carotid Intima-Media Thickness; CAC: Coronary Artery Calcium; MESA: Multi-Ethnic Study of Atherosclerosis; MESA Air: MESA and Air Pollution; NPACT: National Particle Component Toxicity Initiative; PM2.5: Particulate matter <2.5μm in aerodynamic diameter; EC: Elemental Carbon; OC: Organic Carbon; IQR: Interquartile Range; IDW: Inverse Distance Weighting.

## Competing interests

The authors declare that they have no completing interests.

## Authors’ contributions

MS carried out the statistical analysis and wrote the draft of the manuscript; JDK assisted in the study design and in drafting the manuscript; SYK assisted with the data analysis; TLV helped design the monitoring network and planned the pollutant laboratory analyses; TRG coordinated the pollutant laboratory analyses; JFP helped design the ultrasound examinations and performed the ultrasound readings; MJB helped design the CT scan examinations; AVDR assisted in study design and analysis; SV assisted in the study design, supervised the data analysis and helped to draft the manuscript. All authors read and approved the final manuscript.
